# The *OsMPK15* Negatively Regulates *Magnaporthe oryza* and *Xoo* Disease Resistance via SA and JA Signaling Pathway in Rice

**DOI:** 10.3389/fpls.2019.00752

**Published:** 2019-06-21

**Authors:** Yongbo Hong, Qunen Liu, Yongrun Cao, Yue Zhang, Daibo Chen, Xiangyang Lou, Shihua Cheng, Liyong Cao

**Affiliations:** ^1^State Key Laboratory of Rice Biology, China National Rice Research Institute, Hangzhou, China; ^2^Zhejiang Key Laboratory of Super Rice Research, China National Rice Research Institute, Hangzhou, China

**Keywords:** *OsMPK15*, *PRs*, SA/JA, ROS, *M. oryzae*, *Xoo*, rice

## Abstract

Mitogen-activated protein kinase (MAPK) cascades play central roles in response to biotic and abiotic stresses. However, the mechanisms by which various MAPK members regulate the plant immune response in rice remain elusive. In this article, to characterize the mechanisms, the knock-out and overexpression mutants of *OsMPK15* were constructed and the disease resistance was investigated under the various fungal and bacterial inoculations. The knock-out mutant of *OsMPK15* resulted in the constitutive expression of pathogenesis-related (*PR*) genes, increased accumulation of reactive oxygen species (ROS) triggered by the pathogen-associated molecular pattern (PAMP) elicitor chitin, and significantly enhanced the disease resistance to different races of *Magnaporthe oryzae* and *Xanthomonas oryzae* pv. *oryzae* (*Xoo*), which cause the rice blast and bacterial blight diseases, respectively. On contrary, the expression of *PR* genes and ROS were down-regulated in the *OsMPK15*-overexpressing (OsMPK15-OE) lines. Meanwhile, phytohormones such as salicylic acid (SA) and jasmonic acid (JA) were accumulated in the *mpk15* mutant lines but decreased in the OsMPK15-OE lines. The expression of SA- and JA-pathway associated genes were significantly upregulated in the *mpk15* mutant, whereas it was down regulated in the OsMPK15-OE lines. We conclude that *OsMPK15* may negatively regulate the disease resistance through modulating SA- and JA-mediated signaling pathway.

## Introduction

The plant has evolved two-layered immune systems to fight off the pathogen attacks, including pattern-recognition receptors (PRRs) triggered immunity (PTI) and specific pathogen-derived effectors triggered immunity (ETI) that is activated by recognition of cytoplasmic resistance proteins (Monaghan and Zipfel, 2012). Perception of extracellular signals, transmission into a cell, and the subsequent activation of defense response via phosphorylation play a central role in defense signaling (Yang et al., 1997). Among them, the mitogen-activated protein kinase (MAPK) cascade is one of the well-characterized and conserved signaling pathways that play vital importance in plant growth and development as well as abiotic and biotic stress response (Hirt, 1997; Yang et al., 1997; Zhang and Klessig, 2001).

Plant MAPK cascade signaling modules consist of three functionally intertwined protein kinases including MAPK kinase kinase (MPKKK), MAPK kinase (MPKK), and MAPK. The basic process is that MPKKK phosphorylate and activate MPKKs, which in turn phosphorylate and activate MAPKs. The activated MAPKs are commonly imported into nucleus, and further interact with specific downstream components such as transcription factors (Tena et al., 2001; Asai et al., 2002; Koo et al., 2009; Jalmi and Sinha, 2016). Until now, at least 17 rice MAPKs have been identified according to *in silico* search of rice genome database but still a large number of members remain yet to be characterized (Reyna and Yang, 2006).

With increase in number of MAPKs characterized, rice, as a monocotyledonous model plant, is subjected to study the roles of MAPKs in signaling pathway. The first characterized rice MAPK is *OsBWMK1* (*OsMPK12*), which was induced by *Magnaporthe oryzae* and mechanical wounding. OsBWMK1 protein contains TDY motif and has a kinase activity that mediates salicylic acid (SA)-dependent defense responses by phosphorylating and activating the target transcription factor *OsWRKY33* and *OsEREBP1*, resulting in high levels of pathogenesis-related (*PR*) transcript (He et al., 1999; Cheong et al., 2003; Koo et al., 2009). Furthermore, overexpression of *OsMAPK12-1* (a transcript of *OsBWMK1*) inhibited seed germination and seedling growth, and enhanced disease resistance to *Xoo.* *PXO099* and *Xanthomonas oryzae* pv. *oryzicola* (*Xoc*) *RS105*; whereas the knockdown lines displayed the opposite phenotype (Xiao et al., 2017). Transcript of *OsBIMK1* will be accumulated upon treatment with benzothiadiazole (BTH) and jasmonic acid (JA), mechanical wounding, or rice-*M. oryzae* interaction (Song and Goodman, 2002). Suppression of abscisic acid (ABA) and pathogen infection induced by *OsMAPK5* may result in a constitutive expression of *PR* genes such as *PR1* and *PR10*, indicating that *OsMAPK5* negatively regulates fungal and bacterial disease resistance (Xiong and Yang, 2003; Xie et al., 2014; Bertini et al., 2018). *OsMAPK6* was characterized to be post-translationally activated in a cell culture by a sphingolipid elicitor and regulated by the small GTPase *OsRac1*, heterotrimeric G-protein, and *OsMPKK10.2*. OsMPKK10.2 phosphorylating and activating OsMAPK3/6 *in vitro*, resulting in enhanced resistance to both *Xoc* resistance and drought tolerance (Ma et al., 2017). By contrast, knockdown (RNAi) of *OsMAPK6* resulted in a reduction of *OsPAL* mRNA transcript (Liebertherr et al., 2005; Yuan et al., 2007). *OsMAPK6* was also essential for the chitin elicitor-induced biosynthesis of diterpenoid phytoalexins from glycolysis to secondary metabolite biosynthesis (Kishi-Kaboshi et al., 2010). Recently, group C member *OsMPK7* is regulated by *OsMKK3*, which interacts with and phosphorylates the target protein OsWRKY33, mediating the resistance against *Xoo* via up-regulation of *PR* genes, cell wall structure maintenance, and cell metabolism (Jalmi and Sinha, 2016).

In addition to its important role in biotic stresses, plant MAPK cascade is also involved in grain size through control cell division (Bent, 2001). Map-based cloning suggested that rice small grain 1 (*smg1*) mutant that encodes OsMKK4, could promote grain size by influencing cell proliferation (Duan et al., 2014). Other examples include the dwarf and small grain 1 (*dsg1*), which exhibits small grains, dwarfism, and erect leaves. *DSG1* encodes a MAPK 6 (OsMAPK6), plants of which have larger grain and sparser panicles than the wild type (WT) plants due to cell differentiation and proliferation. OsMAPK6 strongly interacts with OsMKK4, which promotes cell proliferation and grain size via brassinoseroids (BR) signaling pathway (Liu et al., 2015). *GSN1* encodes the MAPK phosphatase OsMKP1, which interacts with and inactivates the MAPK OsMAPK6 via dephosphorylation. Meanwhile, the suppression of *OsMAPK6*, *OsMKK4*, and *OsMKKK10* resulted in smaller grains and dense panicles (Guo et al., 2018).

Although many studies on the MAPK cascades in immune response have been conducted in Arabidopsis and rice, those work are mainly focused on the TEX motif members such as the *OsMAPK3*/*6*, *OsMPK4*, and *OsMPK5* mediated signaling pathway. There are 17 identified MAPKs members in rice genome, of which 11 members contain the TDY phosphorylation site and six members have the TEY motif. They could be divided into six groups by phylogenetic analysis, of which *OsMPK15* belongs to group F (TDY type) with a length of 498 amino acid. Previous studies found that the transcript of *OsMPK15* was suppressed by virulent blast race earlier than 24 h while it was accumulated 48 h after inoculated with the avirulent *M. oryzae* race (Reyna and Yang, 2006). Little is known about the mechanisms of *OsMPK15* in the defense response against *M. oryzae* and *Xoo*. In our study, the Crispr/Cas9-edited mutagenesis and overexpression approaches were performed to characterize the function of *OsMPK15* in disease resistance. We found that *OsMPK15* acted as a negative regulator in defense response to the *M. oryzae* and *Xoo* pathogens. We also found that the knock-out of *OsMPK15* significantly increased grain length.

## Materials and Methods

### Plant Materials Generation and Growth Conditions

A *mpk15* mutant was generated using Crispr/Cas9 editing system by Biogle company (Hangzhou, China). The target sequence of *OsMPK15* (LOC_Os11g17080) was designed TTCCTCTATCAGTTGCTTCG**AGG**. The *mpk15* mutant lines were homozygous through sequence confirmation. The full length ORF of *OsMPK15* was cloned into a modified pCAMBIA 1301 vector under the control of maize ubiquitin promoter, yielding the pCAMBIA1301-Ubi::OsMPK15 plasmid. The *OsMPK15*-overexpressing (OsMPK15-OE) lines were generated by introducing the yielded plasmid to the rice cultivar ZH11. Transgenic rice plants were grown in a controlled field under natural conditions.

### Pathogen Inoculation

To evaluate the rice blast disease resistance at the seedling stage, we used punch inoculation on 30-day-old seedling rice leaves (Park et al., 2012). *M. oryzae* isolates were grown on oat agar medium for 10 days. Spores were collected and the concentration was adjusted to 10^5^ conidia /ml supplemented with 0.02% tween-20 before inoculation. Punch inoculation of detached leaves was carried out as follows: dip 5 μl spore suspension for each drop using pipette tip at three spots on each leaf. The inoculated leaves were kept in the dark for 24 h at 25°C with 100% humidity and then moved to the growth chamber under the 25°C, 12 h light/12 h dark cycle normal condition. Lesion length was measured at 6 days post-inoculation (dpi). Relative quantification of fungal DNA amount was calculated using qRT-PCR method by assessing the genomic DNA level of a *M. oryzae 28S rDNA* and relative fungal growth was presented as ratios obtained by comparison of the genomics fungal *28S rDNA* levels with a rice *OsEF1* genomic DNA levels according to the previous report (Hong et al., 2017).

To evaluate resistance of rice bacterial blight disease, Philippine *Xoo* strain *PXO96*, and Chinese *Xoo* strain *Zhe817* were grown in NA media. Rice plants at the booting stage were inoculated using the leaf-clipping method (Sun et al., 2004; Hong et al., 2017) and the inoculated plants were moved to a greenhouse under controlled conditions at 30°C in daytime and 25°C in the night and maintained suitable humidity. Disease phenotype was recorded by measuring the lesion length at 15 dpi.

### Measurement of ROS

Leaf disks from 3-month-old plants were cut and preincubated in sterile distilled water overnight. ROS generation after chitin treatment was measured using the luminol chemiluminescence assay (Park et al., 2012). Briefly, three-leaf disks per sample were placed in a 1.5 ml microcentrifuge tube containing 100 μl luminol (L-012 from Wako Pure Chemical Corporation instead, CAS. No. 143556-24-5), 1.0 μl horseradish peroxidase (Sigma), and 1.0 μl elicitor (800 nM Chitin, water as a mock control) and the luminescence readout was recorded every 10 s for 20 min in Glomax 20/20 Luminometer (Promega). At least three biological replications were performed for each sample.

### SA and JA Quantification

Quantification of SA and JA was carried out according to the method described previously (Fu et al., 2012; Hong et al., 2017). Quantification of SA and JA was performed by an HPLC-MS system (Model 1290/6460, Agilent) with stable isotope-labeled SA and JA as an internal standard. Each measurement was replicated three times.

### Gene Expression Analysis by Real-Time PCR

Total RNA of the wild-type, *mpk15* mutant, and OsMPK15-OE lines at the same developmental stage were extracted using the TRIzol reagent (Invitrogen, Shanghai, China). First-strand cDNA was synthesized using PrimeScript RT reagent Kit with gDNA eraser (TaKaRa, Dalian, China) according to the manufacturer’s instructions. 0.5 μl cDNA was used for a template of qRT-PCR with a LightCycler 480 II real-time PCR system (Roche, United States). The *OsActin* gene (LOC_Os03g50885) was used as an internal control. Gene-specific primers are listed in Supplementary Table S1. Three independent biological and technical replicates were run for each expression assessment.

### Protein-Protein Interaction Prediction

Protein-protein interaction could be mined from the online resources such as STRING 10.5 database^1^. Briefly, the full amino acid sequence and organism of OsMPK15 were submitted for a query, and the predicted computationally scored protein and protein interaction network could be obtained.

### Evaluation of Agronomic Traits

Plant height and tiller number for different rice lines were examined under the field conditions. Harvested grains were air-dried and stored at room temperature for 1 month. Twenty random main panicles from each line were chosen for measuring the panicle weight, the total number of grains per panicle, seed setting rate, and grain yield per plant using the conventional methods. 1,000-grain weight and grain length and width from the WT, *mpk15* mutant, OsMPK15-OE plants were calculated using an automatic seed analysis system (WanShen SC-G, Hangzhou, China). The measurements were repeated at least three times and the repeated data were averaged for analysis.

### Statistical Analysis

The two-sample student’s *t*-test was used to compare the difference in mean and a *p*-value of ≤ 0.05 was considered significant.

## Results

### Generation of CRISPR/Cas9 Edited *mpk15* Mutant and OsMPK15-OE Lines

**FIGURE 1:**
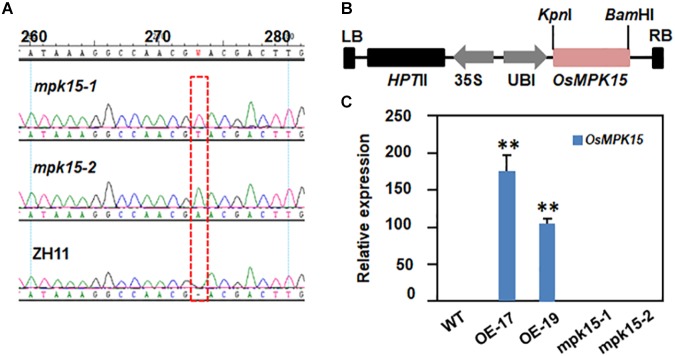
Generation of Crispr/Cas9 edited *mpk15* mutant and OsMPK15-OE lines. **(A)** Sequence confirmation of the homogenous *mpk15-1* and *mpk15-2* mutant lines., **(B)** Schematic diagram of the overexpression construct for rice transformation. HPT II, Hygromycin phosphotransferase II; LB, left border; RB, right border; Ubi, maize ubiquitin promoter; 35S, CaMV 35S promoter. **(C)** *OsMPK15* expression levels in the WT, *mpk15* mutant, and OsMPK15-OE lines at 30-days-old seedling. Data presented are the means ± SD from three independent experiments. ^∗^*P* ≤ 0.05; ^∗∗^*P* ≤ 0.01 (Student’s *t*-test).

**FIGURE 2:**
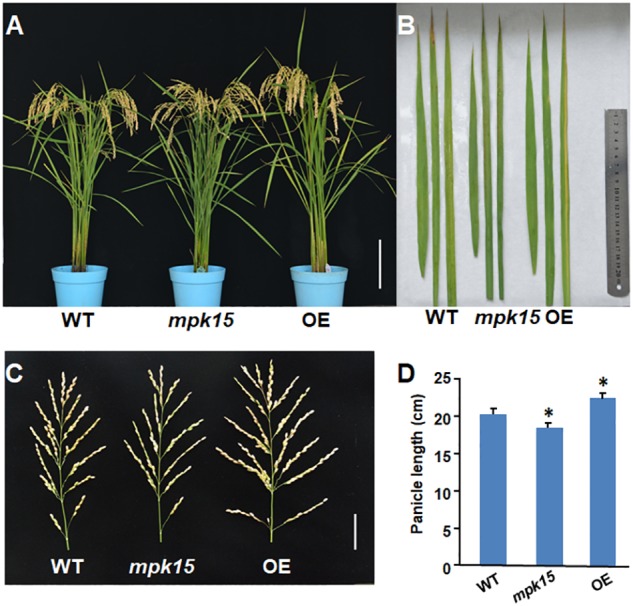
Phenotypic comparison of the WT, *mpk15* mutant, and OsMPK15-OE lines. **(A)** The phenotype of the WT, *mpk15* mutant, and OsMPK15-OE lines at 102 days post-sowing (dps). Bars indicate a length of 15 cm. **(B)** Magnified view of the flag leaf, top second, and top third leaves in the WT, *mpk15* mutant, and OsMPK15-OE line at 102 dps. Among them, the top third of OsMPK15-OE has obvious bacterial blight lesion. **(C,D)** panicle phenotype and length in the WT, *mpk15* mutant, and OsMPK15-OE lines. Bars indicate 5 cm. Data presented are the mean ± SD from three independent experiments. ^∗^*P* ≤ 0.05; ^∗∗^*P* ≤ 0.01 (Student’s *t*-test).

To better understand the molecular mechanism of rice MAPK family underlying plant-microbe interaction and immune response, we explored the expression pattern of *OsMPK15* in response to *M. oryzae* infection and phytohormone treatment. Since previous studies have pointed to the conclusions that the transcript of *OsMPK15* was suppressed by virulent blast race earlier than 24 h while it was accumulated 48 h after inoculated with the avirulent race (Reyna and Yang, 2006) and that the transcript of *OsMPK15* was also accumulated by JA treatment (Reyna and Yang, 2006), we therefore hypothesized that *OsMPK15* may be involved in the disease resistance. To test this hypothesis, we generated both the *mpk15* mutant and OsMPK15-OE lines using the reverse-genetic approach. Sequencing analysis demonstrated that the two *mpk15* mutant lines were homozygous, suitable for the proposed investigation, which inserted “A” or “T” resulting in a shift mutation, respectively (Figure 1A). For overexpression of *OsMPK15*, the full coding sequence of *OsMPK15* was constructed into a modified binary vector under the control of maize ubiquitin promoter (Figure 1B) and transformed into the rice cultivar ZH11. We selected two over-expressing lines, the transcript of *OsMPK15* in OE-17 and OE-19 was 173.1 and 104.5 times higher than the WT by qRT-PCR (Figure 1C). During our study, we did not observe any significant changes in morphological and developmental phenotypes between the OsMPK15-OE and WT plants at the seedling and adult stages (Figure 2A). On the flag, top second, and top third leaves, we found that slightly lesion mimic was found in the WT while there was no lesion mimic in the *mpk15* mutant under the field condition. However, increased susceptibility to bacterial blight was observed in the top third leaf of OsMPK15-OE line (Figure 2B).

### The Role of *OsMPK15* in Disease Resistance to *M. oryzae*

**FIGURE 3:**
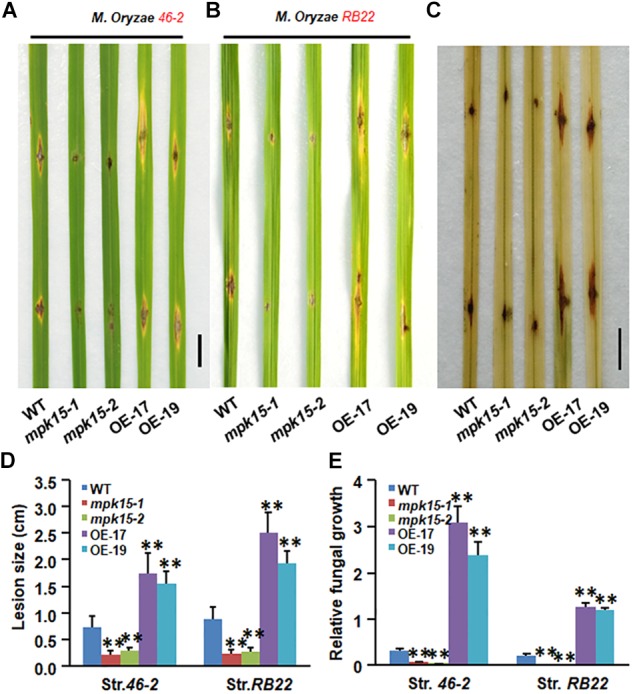
*OsMPK15* negatively regulates *M. oryzae* disease resistance in detached leaves. **(A,B)** Phenotype of the at 30-days-old WT, *mpk15* mutant, and OsMPK15-OE plants inoculated by the punch method with spore suspension (1 × 10^5^ per ml) of *M. oryzae* strain *46-2* or race *RB22* at 6 dpi. Bars indicate 1 cm. **(C)** DAB staining in *M. oryzae* strain *RB22* inoculated leaves of WT, *mpk15* mutant, and OsMPK15-OE plants at 6 dpi. Bars indicate 1 cm. **(D)** Disease lesion size statistics of *M. oryzae* strain *46-2* inoculated WT, *mpk15* mutant, and OsMPK15-OE plants. At least 30 plants in each experiment were evaluated for lesion size with three replicates. **(E)** Quantification of *M. oryzae* growth in strain *46-2* and *RB22*-inoculated leaves of the WT, *mpk15* mutant, and OsMPK15-OE plants at 6 dpi. Amounts of *M. oryzae* *28S rDNA* and rice *OsEF1* genomic DNA were estimated by qRT-PCR and relative fungal growth were shown as ratios of *Mo28S*/*OsEF1*. Three independent experiments were performed with similar results in **(A**–**E)** Data presented in **(D,E)** are the mean ± SD from three independent experiments. ^∗^*P* ≤ 0.05; ^∗∗^*P* ≤ 0.01 (Student’s *t*-test).

To investigate the role of *OsMPK15* in rice innate immunity, we first examined whether *OsMPK15* is responsible for resistance to rice blast at the seedling stage. Two *M. oryzae* compatible strains, *46-2* and *RB22*, were used to inoculate the 30-days-old seedling of *mpk15* mutant, OsMPK15-OE and non-transgenic WT seedlings by the punch inoculation and disease levels were evaluated by the lesion diameter and the *in planta* fungal growth. As shown in Figure 3A,B, the overall blast disease phenotype on the OsMPK15-OE lines OE-17 and OE-19 was more severe than those in the non-transgenic WT plants after inoculation with strains *46-2* and *RB22*. Typical blast lesions were seen on leaves of OsMPK15-OE and non-transgenic WT plants, whereas almost no typical blast lesion was observed on the leaves of *mpk15* mutant lines (Figure 3A,B). At 6 dpi of *M. oryzae*, the average blast lesion sizes on the inoculated leaves of OsMPK15-OE plants were increased by 2.14 to 2.41 folds while the lesion size on the inoculated leaves of the *mpk15* mutant plants were decreased by 60.5 to 68.9%, as compared with that in the WT plants (Figure 3D). Next the *in planta* fungal growth, measured by the genomic DNA level of the *28S rDNA* gene of *M. oryzae*, indicated that OsMPK15-OE plants had more growth of *M. oryzae* in the inoculated leaves, increased by 7.60 to 9.92-folds, whereas the *mpk15* mutant plants decreased by 81.71 to 89.15%, as compared with that in the WT plants (Figure 3E). Taken together, these results demonstrated that the *mpk15* mutant plants exhibited an increased resistance while OsMPK15-OE displayed increased susceptibility to *M. oryzae*.

### *OsMPK15* Negatively Regulates Resistance to *X. oryzae* pv. *oryzae*

**FIGURE 4:**
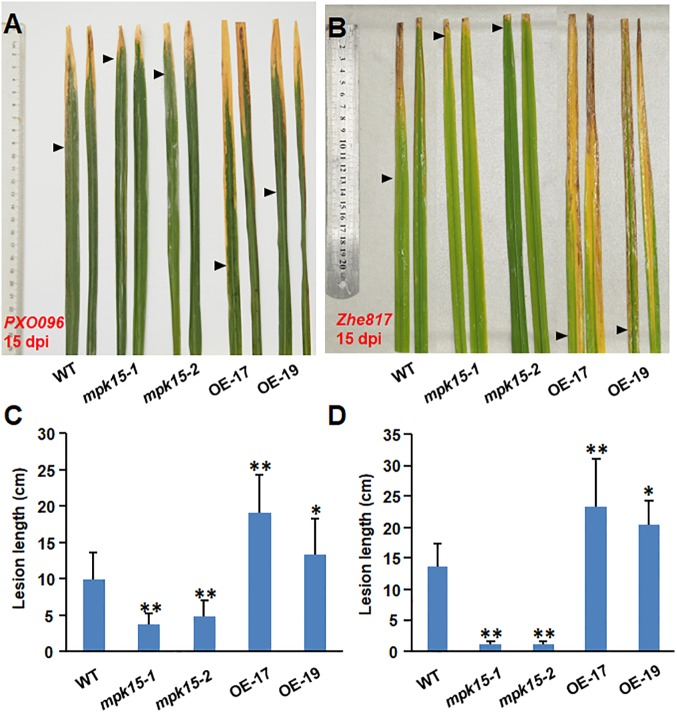
*OsMPK15* negatively regulates the bacterial disease resistance to *X. oryzae* pv. *oryzae*. The WT, *mpk15* mutant, and OsMPK15-OE plants were inoculated with *Xoo* strains *PXO96* and *Zhe817* using the leaf clipping method at the 90-days-old (booting stage). **(A,B)** Disease symptom on the *PXO96* and *Zhe817*-inoculated leaves at 15 dpi. Rulers were indicated on the left. **(C,D)** Lesion length on the inoculated leaves at 15 dpi. At least 12 plants in each experiment were used for measurement of the lesion length. Data presented in **(C,D)** are the mean ± SD from three independent experiments. ^∗^*P* ≤ 0.05; ^∗∗^*P* ≤ 0.01 (Student’s *t*-test).

Rice bacterial leaf blight, caused by *Xoo*, is also one of the most devastating diseases threating rice production. We then evaluated the resistance of the OsMPK15-OE and *mpk15* mutant lines against *Xoo* by leaf-clipping inoculating of adult plants at 3-month-old plants with two *Xoo* strains, *PXO96* and *Zhe817*, and the disease severity was evaluated by measuring lesion length at 15 dpi. As shown in Figure 4A,B, the overall *Xoo*-caused blight disease on the *mpk15* mutant lines was less severe while the OsMPK15-OE plants were more severe, as compared with those in WT plants. The average length of the blight lesions on the *PXO96* strain inoculated flag leaves of the *mpk15* mutant plants were 3.68 and 4.81 cm, representing the reductions of 51.47 and 62.82%, while those of the OsMPK15-OE plants were 18.98 and 13.32 cm, indicating the increases of 34.45 and 91.58%, as compared with that (9.90 cm) in the WT plants (Figure 4C). The similar, even more evident, a pattern was observed in Figure 4D, as inoculated with *Zhe817* strain. These results indicate that the *mpk15* mutant lines exhibited an increased resistance while the OsMPK15-OE lines displayed increased susceptibility to *Xoo*.

### Chitin-Induced ROS Accumulation in the *mpk15* Mutant Lines

**FIGURE 5:**
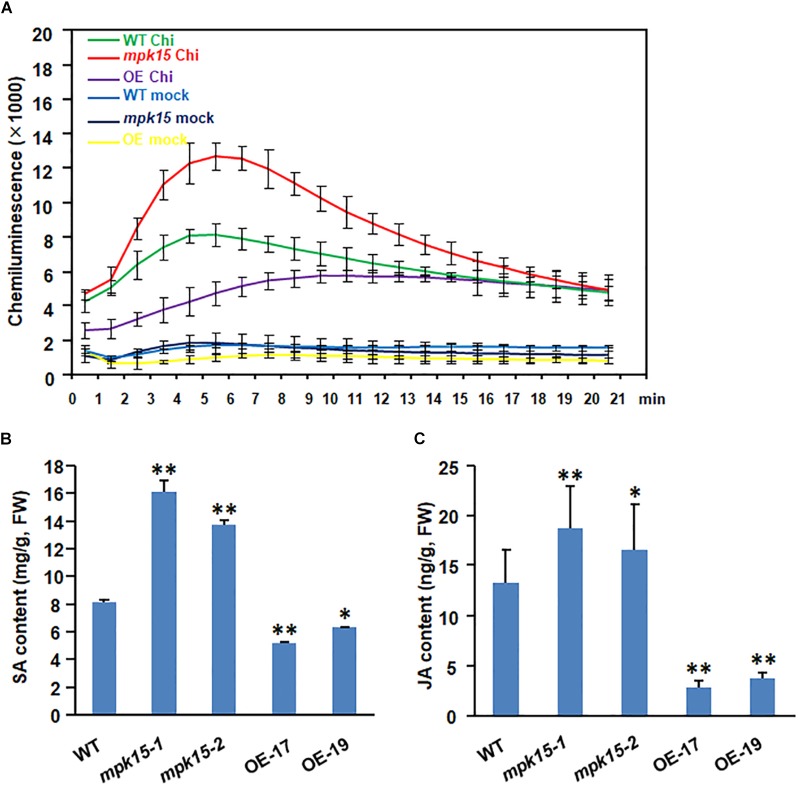
Increase in the ROS accumulation and disease-related hormone levels in the *mpk15* mutants. **(A)** ROS accumulation dynamics in the WT, *mpk15* mutant, and OsMPK15-OE plants at 90 dps after treatment of chitin and water (mock) treatment served as a control. Error bars are computed from three replicates (*n* = 3). **(B,C)** The 30-day-old seedling leaf samples of the WT, *mpk15* mutant and OsMPK15-OE lines under normal conditions were used for analyses of SA and JA contents. FW: fresh weight. Data presented are the mean ± SD from three independent experiments. ^∗^*P* ≤ 0.05; ^∗∗^*P* ≤ 0.01 (Student’s *t*-test).

To investigate whether *OsMPK15* can suppress the plant PTI response, we used chemical luminescence to monitor the dynamics of ROS bursts in the *mpk15* mutant, OsMPK15-OE, and WT plants treated with chitin, which is recognized by OsCEBiP/OsCERK1 in rice (Kaku et al., 2006; Shimizu et al., 2010; Park et al., 2012). The data revealed that the ROS accumulation in the *mpk15* mutant lines was significantly higher than that in WT in response to chitin treatments while the accumulation in the OsMPK15-OE lines was significantly lower than that in WT. The peak ROS level occurred about 5 and 6 min after the chitin treatment (Figure 5A). The chitin treated ROS accumulation in the *mpk15* mutant lines was 1.56 times greater than that in WT, whereas the OsMPK15-OE lines had only 58.3% of the WT plants at the peak time (Figure 5A). In contrast, the water controls of different lines all were kept the basal ROS level. This finding was also confirmed by quantification of the endogenous H_2_O_2_ using 3,-3′-diaminobenzidine (DAB) staining (Figure 3C). These results suggested that the chitin-triggered ROS accumulation is suppressed in OsMPK15-OE lines and increases in the *mpk15* mutant lines.

### SA and JA Accumulation in the *mpk15* Mutant Lines

Salicylic acid and jasmonic acid are the most important plant hormones that play vital roles in regulating immune responses (Qiu et al., 2007; Koo et al., 2009; Pieterse et al., 2012; Yang et al., 2012; Yang et al., 2013; Liu et al., 2016). To further investigate whether these pathways are involved in the *OsMPK15*-mediated defense signaling pathway, we then checked the endogenous SA and JA levels in the 4-week-old OsMPK15-OE, *mpk15* mutant, and WT plants under normal conditions. The SA levels in the *mpk15* lines were 1.69 to 1.98-folds higher than those in the non-transgenic WT plants while the OsMPK15-OE lines had reduced 36.69 and 22.06% as compared with the WT plants, respectively (Figure 5B). Similarly, the endogenous JA levels in the *mpk15* mutant lines were increased by 41.47and 24.06% while the OsMPK15-OE lines were reduced by 78.87 and 71.91% as compared with WT plants, respectively (Figure 5C). These results suggested that the SA and JA accumulation was reduced in the OsMPK15-OE lines and increased in the *mpk15* lines under normal conditions.

**FIGURE 6:**
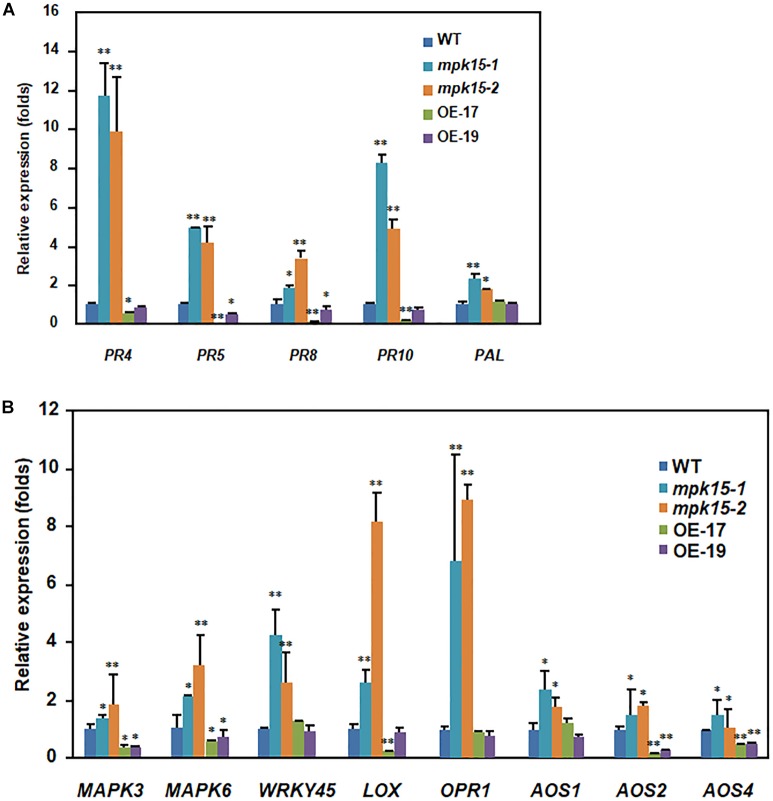
Expression pattern of defense signaling genes in the WT, *mpk15* mutant, and OsMPK15-OE lines. The 30-day-old seedlings samples of different lines under normal conditions were used for qRT-PCR analyses. **(A)** The expression pattern of pathogenesis-related (*PR*) genes includes *PR4*, *PR5*, *PR8*, *PR10*, and *PAL*. **(B)** Expression pattern of the *MPK3*, *MPK6*, SA-mediated signaling marker gene *WRKY45*, and JA biosynthesis genes include *LOX*, *OPR1*, *AOS1*, *AOS2*, and *AOS4*. Data presented are the mean ± SD from three independent experiments. ^∗^*P* ≤ 0.05; ^∗∗^*P* ≤ 0.01 (Student’s *t*-test).

Improved resistance to *M. oryzae* and *Xoo*, increased the accumulation of chitin-triggered ROS, and elevated SA and JA levels in the *mpk15* mutant lines motivated us to examine whether the *mpk15* mutant alters the expression of defense genes. We analyzed the expression patterns of *PR4*, *PR5*, *PR8*, *PR10*, and *PAL* in the 4-week-old *mpk15* mutant, OsMPK15-OE, and WT plants under normal conditions. As shown in Figure 6A, the expression levels of *PR4*, *PR5*, *PR8*, *PR10*, and *PAL*, of which *PAL* is a SA biosynthesis gene, were markedly upregulated in the *mpk15* mutant lines, showing increases of 9.85, 4.15, 3.41, 4.94, and 2.23-folds as compared with the WT plants while keeping at a very lower levels (8.9 to 89% of WT) in the OsMPK15-OE lines. In addition, we also investigated the expression of MAPK central genes including *MAPK3* and *MAPK6*, the SA signaling marker gene *WRKY45* (Shimomo et al., 2007), and the JA biosynthesis genes *LOX*, *OPR1*, *AOS1*, *AOS2*, and *AOS4* (Lee et al., 2001; Feussner and Wasternack, 2002). The *MAPK3*, *MAPK6*, and *WRKY45* were significantly up-regulated in the *mpk15* mutant lines and suppressed in the OsMPK15-OE lines (Figure 6B). In the *mpk15* mutant lines, the JA biosynthesis genes including *LOX* and *OPR1* were markedly up-regulated, showing increases of 2.61 and 6.78-folds higher than those in the WT plants while the other JA biosynthesis genes such as *AOS1*, *AOS2*, and *AOS4* were slightly upregulated, showing increases of 1.46 to 2.36 times higher than those of the WT plants; whereas they were suppressed the in OsMPK15-OE lines (Figure 6B), in good agreement with the elevated SA and JA content in the *mpk15* mutant as shown in Figure 5C,D. These results demonstrated *OsMPK15* might play a negative role in the regulation of SA- and JA-signaling pathway against *M. oryzae* and *Xoo*.

### Protein-Protein Interaction Prediction of OsMPK15

To further search the upstream regulator of *OsMPK15* and the downstream substrate with *M. oryzae* and *Xoo* interaction, we used STRING 10.5 database^2^ for screening possible protein-protein interaction. Bioinformatics prediction suggested that there were 10 proteins to interact with the OsMPK15 protein (Supplementary Figure S1 and Supplementary Table S2). One protein named OsMPKK5 (LOC_Os06g09190) may be the upstream regulator of *OsMPK15*; the other five members were protein phosphatase. We also speculate that PP2C or protein phosphatase may interact with the OsMPK15 protein to dephosphorylate The putative role of MAPK to suppress the defense response (Schweighofer et al., 2007; Sidonskaya et al., 2016), needs to be further confirmed by the yeast two-hybrid or Co-IP methods in the follow-up study.

### Decreased Grain Yield and Increased Grain Length in the *mpk15* Mutant

Interestingly, in addition to the involvement in disease resistance, we also compared the agronomic traits between the *mpk15* mutant, OsMPK15-OE, and WT plants. The plant height of the OsMPK15-OE lines was higher than that in WT (Figure 2A, 7A). Furthermore, the grain number per panicle and panicle length of the OsMPK15-OE line were more than those of WT while the *mpk15* mutant had less grain than WT (Figure 2C,D). We speculated that enhanced disease resistance in the *mpk15* mutant might consume much more energy, leading to less biomass and grain number panicle.

**FIGURE 7:**
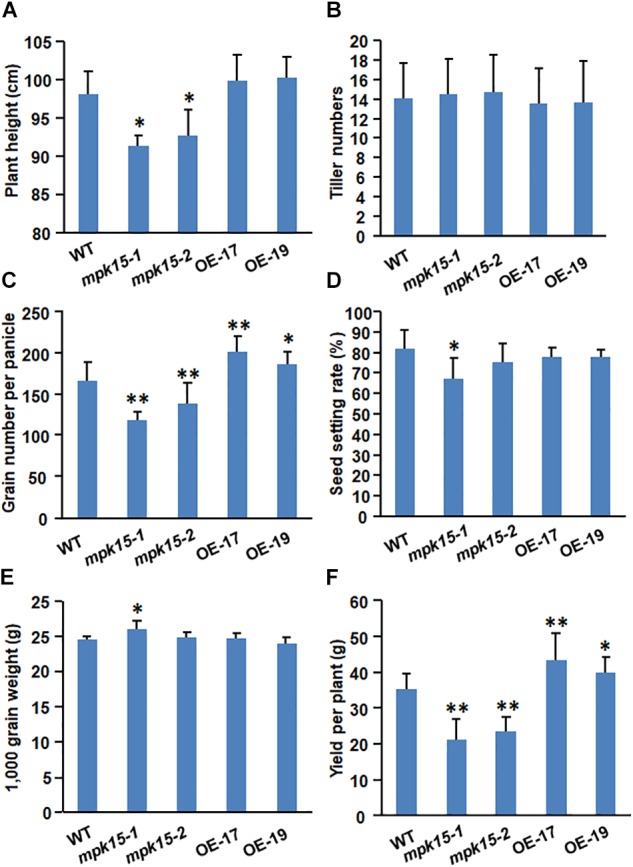
Agronomic phenotypes of the WT, *mpk15* mutant, and OsMPK15-OE lines. **(A,B)** Comparison of plant height and tiller numbers among the WT, *mpk15* mutant, and OsMPK15-OE plants (*n* = 10). **(C–F)** Comparison of grain number per panicle, seed setting rate, 1,000-grain weight, and yield per plant among the WT, *mpk15* mutant, and OsMPK15-OE lines (*n* = 10). Data presented are the mean ± SD from 2 years of independent experiments with a similar result. ^∗^*P* ≤ 0.05; ^∗∗^*P* ≤ 0.01 (Student’s *t*-test).

**FIGURE 8:**
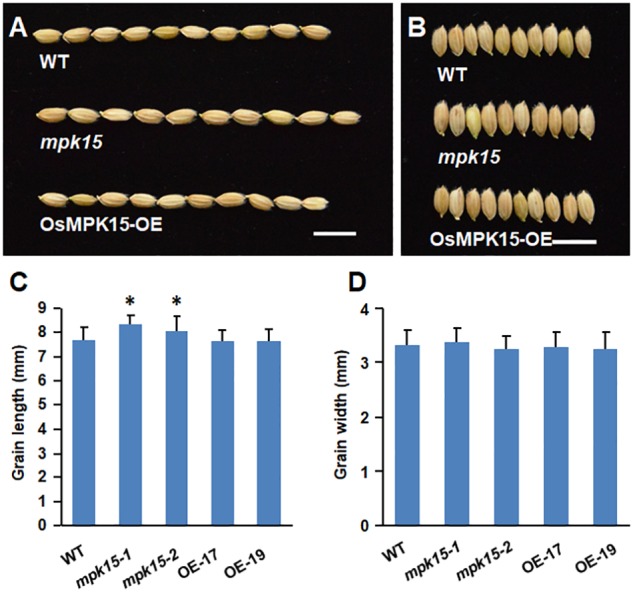
Grain length and width of the WT, *mpk15* mutant, and OsMPK15-OE lines. **(A,B)** The phenotype of grain length and width from the WT, *mpk15* mutant, and OsMPK15-OE lines. Bars indicate 10 mm. **(C,D)** Statistics of grain length and width (*n* = 300), respectively. ^∗^*P* ≤ 0.05; ^∗∗^*P* ≤ 0.01 (Student’s *t*-test).

Compared with the wild-type plants, the *mpk15* mutant lines displayed a marked decrease of grain number per panicle and seed setting rate and increased 1,000-grain weight, respectively; whereas, the OsMPK15-OE lines significantly increased the grain number per panicle (Figure 7C). The tiller numbers, seed setting rate, and 1,000-grain weight were not significantly different between OsMPK15-OE and WT (Figure 7B,D,E). Our field assessments of grain yield per plant showed that the OsMPK15-OE lines had an increased grain yield per plant of 14.02 and 23.23% compared with the wild-type plant whereas the *mpk15* mutant lines were only 60.2 to 66.93% of the WT plants, respectively (Figure 7F). In addition, the knock-out of *OsMPK15* produces larger seeds while no significant differences were observed in grain length, width, and seedling development between the OsMPK15-OE and WT plants (Figure 8A–D). These results demonstrated that *OsMPK15* may positively regulate grain yield production by sacrificing disease resistance.

## Discussion

Rice durable and broad-spectrum disease resistance is a trait of agronomic importance for stable high yield variety. Rice MAPK cascade is one of the evolutionarily conserved signaling pathways in regulating defense response (Zhang and Klessig, 2001). Although substantial progress has been made, however, most of the rice MAPKs studies were focused on the “TEY” motif group such as *OsMAPK3*/*6*, whereas the “TDY” group such as group E remains uncharacterized. In the present study, we generated the *mpk15* mutants and OsMPK15-OE lines and further provided the evidence of group E member of MAPK, *OsMPK15* negatively regulating *M. oryzae* and *Xoo* disease resistance.

### OsMPK15 Is a Switch in Response to *M. oryzae* and *Xoo* Infection

Knock-out of *OsMPK15* lines enhanced disease resistance to different races of *M. oryzae* and *Xoo* while OsMPK15-OE lines significantly increased susceptibility as compared to WT plants (Figure 3, 4). We also found that the transcript of *PR* genes, *OsMAPK3*/*6* and SA/JA signaling pathway associated genes were elevated in the *mpk15* lines, while those were suppressed significantly in the OsMPK15-OE lines (Figure 6), leading to a relatively high level of SA and JA in the *mpk15* lines (Figure 5C,D), implying that *OsMPK15* negatively regulates disease resistance. These results demonstrate that *OsMPK15* functions as an inhibitor to suppress the *OsMAPK3*/*6* cascade signaling pathway in the normal growth condition, which is essential to activate the defense response (Liebertherr et al., 2005; Kishi-Kaboshi et al., 2010; Ma et al., 2017). Allocation of the resources and energy from growth and development to defense response in case of pathogen infection may cause less biomass and low yield (Figure 7F).

Salicylic acid and JA are important hormones in response to biotic stress response. Activation of ETI and systemic acquired resistance often induces the biosynthesis of SA (Century et al., 1997; Shapiro and Zhang, 2001). SA/BTH-induced *OsWRKY45* was regulated by the MAPK-dependent phosphorylation in one of the two branches of the rice SA pathway (Nakayama et al., 2013; Ueno et al., 2013). The involvement of defense responses of the *mpk15* mutant is partially supported by the up-regulation of *PRs* such as *OsPR10* (Figure 6A), which was modulated by the SA signaling pathway (Mitsuhara et al., 2008), indicating that the knock-out of *OsMPK15* could activate the SA signaling pathway, which is in agreement with the previous report that *AtMPK4* and *OsMAPK5* negatively modulate the *PR* gene expression and broad-spectrum resistance (Petersen et al., 2000; Xiong and Yang, 2003). We also observed a high level of JA in the *mpk15* mutant accompanied by an increased expression of the JA biosynthesis genes such as *LOX*, *OPR1*, and *AOS 1*/*2*/*4* (Figure 6B). In addition to induced expression of *PR* genes such as *OsPR5* (Figure 6A), the knock-out of *OsMPK15* could activate JA signaling pathway by upregulating the expression of JA biosynthesis and signaling genes (Mitsuhara et al., 2008). Generally, a high level of JA may repress vegetative growth and affect male fertility. The relatively lower seed setting rate (Figure 7D) in the *mpk15* lines may be attributed to a high level of JA, which is consistent with the previous report (Hou et al., 2010; Yang et al., 2012).

### *OsMPK15* Positively Regulates Yield

There is a general consensus that improved plant disease resistance is usually accompanied by a decrease in growth and yield (Ning et al., 2017). Together with the increased broad-spectrum resistance in the *mpk15* lines, the yield per plant was dramatically reduced compared with the WT plants (Figure 7F). These results indicate that the *mpk15* lines will allocate the energy into defense against different pathogens, resulting in low yield and biomass. By contrast, the OsMPK15-OE plants will transfer more flow from defense response to growth and development. These results suggest that the increased broad-spectrum disease resistance in the *mpk15* mutant came at the penalty of yield losses, which is supported by the previous reports (Jorgensen, 1992; Brown, 2002; Sharp et al., 2002).

We also found that the grain length was dramatically increased and reduced in the *mpk15* and OsMPK15-OE lines, respectively (Figure 8C). In the previous study, activation of *OsMAPK6* and its upstream regulator *OsMKK4* positively influence rice grain size via cell proliferation of brassinosteroids (BRs) signaling and homeostasis (Duan et al., 2014; Liu et al., 2015). By contrast, the deactivation of OsMAPK6 by the MAPK phosphatase 1 (OsMKP1) also leads to smaller grains (Xu et al., 2018). In our study, the transcript of *OsMAPK6* was accumulated in the knock-out of *OsMPK15* lines (Figure 6B), leading to longer grain length in the *mpk15* lines, which was consistent with the former studies (Liu et al., 2015), indicating *OsMPK15 acting* as an inhibitor for grain length.

## Conclusion

In conclusion, we demonstrate that *OsMPK15* negatively regulates the disease resistance to different races of *M. oryzae* and *Xoo* through modulating expression of *PRs* and SA/JA-associated genes as well as ROS burst. Furthermore, the knock-out of *OsMPK15* also results in long grain size.

## Gene Accession Numbers

Sequence data from this study can be found in the GenBank/EMBL data libraries with the following accession numbers: *OsMPK15* (LOC_Os11g17080), *OsPR4* (LOC_Os11g37970), *OsPR5* (LOC_Os12g43380), *OsPR8* (LOC_Os10g28080), *OsPR10* (LOC_Os12g36880), *OsPAL* (LOC_Os02g41680), *OsMAPK3* (LOC_Os03g17700), *OsMAPK6* (LOC_Os06g06090), *OsWRKY45* (LOC_Os05g25770), *OsLOX* (LOC_Os08g39840), *OsOPR1* (LOC_Os06g11290), *OsAOS1* (LOC_Os03g55800), *OsAOS2* (LOC_Os03g12500), *OsAOS4* (LOC_Os02g12690), *OsActin* (LOC_Os03g50885).

## Author Contributions

YH conceived, designed and performed the experiments and drafted the manuscript. YH and QL analyzed the data. YC and YZ help my plant materials in the field condition, XL has a critical reading to the manuscript and made important suggestions. All authors discussed the results, provided critical feedback, and contributed to the final version of the manuscript.

## Conflict of Interest Statement

The authors declare that the research was conducted in the absence of any commercial or financial relationships that could be construed as a potential conflict of interest.

## Footnotes

1 http://string-db.org

2 http://string-db.org
