# Supplementary material for: The OsMPK15 Negatively Regulates Magnaporthe oryza and Xoo Disease Resistance via SA and JA Signaling Pathway in Rice
Source: Front Plant Sci. 2019 Jun 21;10:752. doi: 10.3389/fpls.2019.00752 (PMC6598650; doi:10.3389/fpls.2019.00752)
Supplement: Supplementary file 1 [file Data_Sheet_1.docx]

**The *OsMPK15* negatively regulates *Magnaporthe Oryza* and *Xoo* disease resistance via SA and JA signaling pathway in rice**

Yongbo Hong^1,2^, Qunen Liu^1,2^, Yongrun Cao^1,2^, Yue Zhang^1,2^, Daibo Chen^1,2^, Xiangyang Lou^1,2,^* , Shihua Cheng^1,2,^*, Liyong Cao^1,2,^*

^1^ State Key Laboratory of Rice Biology, China National Rice Research Institute, Hangzhou 310006, China;

^2^ Zhejiang Key Laboratory of Super Rice Research, China National Rice Research Institute, Hangzhou 310006, China

* **Correspondence:**

**Li-Yong Cao**

National Rice Improvement Center

China National Rice Research Institute, Hangzhou 311400

Tel: 0571-63370329, Fax: 0571-63370265

E-mail: caoliyong@caas.cn

**Shi-Hua Cheng**

National Rice Improvement Center

China National Rice Research Institute, Hangzhou 311400

Tel: 0571-63370235, Fax: 0571-63370265

E-mail: chengshihua@caas.cn

**Xiang-Yang Lou**

State Key Laboratory of Rice Biology

China National Rice Research Institute, Hangzhou 311400

Tel: 0571-63370305, Fax: 0571-63370305

Email: louxiangyang01@caas.cn

*To whom correspondence should be addressed*. E-mail: caoliyong@caas.cn

**ORCID ID: orcid.org/0000-0002-0996-600X**

Table S1. Primers used in this study

| Name | Primer sequence (5’-3’) |
| --- | --- |
| Plasmid constructions | |
| OsMPK15-OE-F | CGGGGTACCCGTCGTCCTCGTTAACTGTCT |
| OsMPK15-OE-R | CGCGGATCCAGGATGGATTTCTTCAGGGGT |
| OsMPK15-GFP-F | GAGCTGTACAAGGGATCCCGTCGTCCTCGTTAACTGTCT |
| OsMPK15-GFP-R | CTTAATTAACTCTCTAGAAGGATGGATTTCTTCAGGGGT |
| mpk15-Cas9-F | TGTGTGTTCTGGAGTCAAGTCGTCGT |
| mpk15-Cas9-R | AAACACGACGACTTGACTCCAGAACA |
| Fungal amount determination | |
| 28S-rDNA-RT-F | TACGAGAGGAACCGCTCATTCAGATAATTA |
| 28S-rDNA-RT-R | TCAGCAGATCGTAACGATAAAGCTACTC |
| eEF1 alpha-RT-F | CAACCCTGACAAGATTCCCT |
| eEF1 alpha-RT-R | AGTCAAGGTTGGTGGACCTC |
| qRT-PCR | |
| OsMPK15-RT-F | CCGTCGTCCTCGTTAACTGTC |
| OsMPK15-RT-R | AGGATGGATTTCTTCAGGGGT |
| OsPR4-RT-F | AGCGCATATTGTGCCACATG |
| OsPR4-RT-R | GGATACACTTGCCACACGAGTCT |
| OsPR5-RT-F | CAACAGCAACTACCAAGTCGTCTT |
| OsPR5-RT-R | CAAGGTGTCGTTTTATTCATCAACTTT |
| OsPR8-RT-F | GTTCATCTGGTCAGCGGATAGC |
| OsPR8-RT-R | TCATAAGTATTATCACGACCGTTCGA |
| OsPR10-RT-F | CCCTGCCGAATACGCCTAA |
| OsPR10-RT-R | CTCAAACGCCACGAGAATTTG |
| OsPAL-RT-F | TCTCGCCATCGCCAACATC |
| OsPAL-RT-R | TGCCCTTGAACCCGTAGTCC |
| OsMAPK3-RT-F | GACGCGAGGAAGTACATGAGG |
| OsMAPK3-RT-R | CAGCGGGTTGAAGGTGAGC |
| OsMAPK6-RT-F | CGCACGCTCAGGGAGATC |
| OsMAPK6-RT-R | GGTATGATATCCCTTATGGCAACAA |
| OsWRKY45-RT-F | TCGTCCGGGAATACGGTGGT |
| OsWRKY45-RT-R | AGGCCTTTGGGTGCTTGGAG |
| OsLOX-RT-F | CCGAGCTTGACGCGAAGA |
| OsLOX-RT-R | GATCGTCGTCGTCCACATTGT |
| OsAOS1-RT-F | CACCGCCGGTCAAAGTCT |
| OsAOS1-RT-R | CCGTATCCGTACAAGCTGATTG |
| OsAOS2-RT-F | CAATACGTGTACTGGTCGAATGG |
| OsAOS2-RT-R | AAGGTGTCGTACCGGAGGAA |
| OsAOS4-RT-F | GAGGAGTACGTGCCGGACAG |
| OsAOS4-RT-R | GGAGTCGTATCGGAGGAAGAGC |
| OsOPR1-RT-F | CGGGAGGAAGGGAACAAGGT |
| OsOPR1-RT-R | AATGGTGCGTCAAGCTCAAAC |

Table S2. List of probable interacting protein of OsMPK15 obtained through *in silico* study using STRING 10.5 database.

| **Name** | **Locus ID** | **Annotation** |
| --- | --- | --- |
| 4350264 | LOC_Os11g17080 | **OsMPK15** |
| LOC_Os01g  36080 | LOC_Os01g36080 | **protein phosphatase 2C containing protein** |
| 4329023 | LOC_Os02g17970 | AGC_PKA/PKG_like.1 - ACG kinases |
| 4328960 | LOC_Os02g16660 | cbxX, chromosomal |
| 4331333 | LOC_Os03g01750 | dual specificity **protein phosphatase** |
| 4340368 | LOC_Os06g09190 | **mitogen-activated protein kinase kinase 5** |
| 4345438 | LOC_Os08g29160 | 5-AMP-activated protein kinase beta-1 subunit |
| 4349934 | LOC_Os11g07850 | **Protein-tyrosine phosphatase** domain containing protein |
| 4350113 | LOC_Os11g11880 | retrotransposon protein, putative, LINE subclass |
| 4351431 | LOC_Os12g03990 | dual specificity **protein phosphatase** |
| 4349934 | LOC_Os12g07590 | **Protein-tyrosine phosphatase** domain containing protein |

FIGURE S1 | List of probable OsMPK15 protein interacting network through using STRING 10.5 database.
