# Supplementary figures and images for: The OsMPK15 Negatively Regulates Magnaporthe oryza and Xoo Disease Resistance via SA and JA Signaling Pathway in Rice
Source: Front Plant Sci. 2019 Jun 21;10:752. doi: 10.3389/fpls.2019.00752 (PMC6598650; doi:10.3389/fpls.2019.00752)

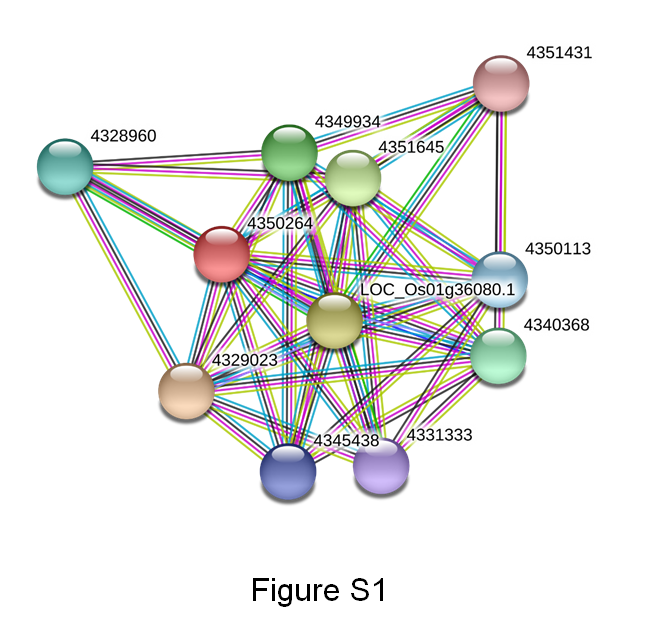

Supplement: Supplementary file 2 [file Image_1.TIF]
